# A Review of Risk Factors for Cognitive Impairment in Stroke Survivors

**DOI:** 10.1155/2016/3456943

**Published:** 2016-05-31

**Authors:** Mohd Faizal Mohd Zulkifly, Shazli Ezzat Ghazali, Normah Che Din, Devinder Kaur Ajit Singh, Ponnusamy Subramaniam

**Affiliations:** ^1^Health Psychology Programme, School of Healthcare Sciences, Universiti Kebangsaan Malaysia, Jalan Raja Muda Abdul Aziz, 50300 Kuala Lumpur, Malaysia; ^2^Center for Neuroscience Services & Research (P3Neuro), Universiti Sains Malaysia (USM), Health Campus, 16150 Kubang Kerian, Kelantan, Malaysia; ^3^Physiotherapy Programme, School of Rehabilitation Sciences, Universiti Kebangsaan Malaysia, Jalan Raja Muda Abdul Aziz, 50300 Kuala Lumpur, Malaysia

## Abstract

In this review, we aimed to identify the risk factors that may influence cognitive impairment among stroke survivors, namely, demographic, clinical, psychological, and physical determinants. A search from Medline, Scopus, and ISI Web of Science databases was conducted for papers published from year 2004 to 2015 related to risk factors of cognitive impairment among adult stroke survivors. A total of 1931 articles were retrieved, but only 27 articles met the criteria and were reviewed. In more than half of the articles it was found that demographical variables that include age, education level, and history of stroke were significant risk factors of cognitive impairment among stroke survivors. The review also indicated that diabetes mellitus, hypertension, types of stroke and affected region of brain, and stroke characteristics (e.g., size and location of infarctions) were clinical determinants that affected cognitive status. In addition, the presence of emotional disturbances mainly depressive symptoms showed significant effects on cognition. Independent relationships between cognition and functional impairment were also identified as determinants in a few studies. This review provided information on the possible risk factors of cognitive impairment in stroke survivors. This information may be beneficial in the prevention and management strategy of cognitive impairments among stroke survivors.

## 1. Introduction

It has been reported that approximately 15 to 30% of stroke survivors live with permanent disability [1]. This includes physical, social, and cognitive functions [2–4]. Various domains of cognitive function such as attention, concentration, memory, social cognition, language, spatial and perceptual skills, and higher-order executive functions may be affected in stroke survivors [5]. The most common cognitive impairments among stroke survivors are memory, orientation, language and attention [6], executive dysfunction (initiation inhibition, mental flexibility), and aphasia [7, 8]. Some of the consequences of cognitive impairments are high prevalence of morbidity and mortality, increased length of hospital stay, increased number of admissions to rehabilitation centre, dependency in ADL, and reduced functional outcomes [9–11].

Cognitive deficits among stroke survivals are widely recognized research area. However, the risk factors for cognitive impairment among stroke survivors have not been addressed adequately [12]. Previous literature reviews predominantly explain vascular risk factors and mechanisms of cognitive impairments [12, 13], impact and treatment options for cognitive impairment [14], and interaction of vascular risk factors with stroke [15], and overlooked or little emphasis was given on the psychological and physical determinants. This may be due to difference in studied variables, methodologies, and outcome measurements. Most of the studies focused on modifiable variables such as sociodemographic and clinical factors. Education level, level of alcohol use, smoking status, dietary intake, atrial fibrillation, and cerebral microbleeds are some of the factors that could be modified to prevent poststroke cognitive decline [16, 17]. Physical activity has been reported to be a protective factor to prevent the risk for cognitive impairment and vascular dementia among older adults with white matter changes [18]. Stroke survivors with vascular cognitive impairment who attended day care rehabilitation were reported to benefit from physical activity and exercise. Exercise is advocated to heighten cognitive function among stroke survivors [19].

Stroke survivors with cognitive impairment are most likely to be dependent in activities of daily living. Further deterioration is possible as a result of limitation in activities. Psychiatric problems are also common among stroke survivors. This may delay recovery process and further impair cognitive function due to adaptations to unhealthy lifestyles or noncompliance to rehabilitation [20]. Prevalence of moderate to severe depression and anxiety was found to be 22.8% and 21.1%, respectively, in stroke survivors [21]. It has been deduced that cognitive factors such as speed of processing and verbal memory were more related to mood disturbances than level of physical independence with a contribution of 51.3% and 38.5% for both depression and anxiety, respectively [21].

In order to improve overall physical function among stroke survivors, emotional disturbances such as depressive symptoms require management because both cognition and mood impairments are associated [21, 22]. Knowledge of the risk factors for cognitive impairments in stroke survivors may be useful in the holistic management. Thus, the aim of this review was to identify risk factors that include demographic, clinical, psychological, and physical determinants that may likely influence cognitive function among stroke survivors.

*(1) Cognitive Impairment in Stroke Survivors*. Cognitive impairment is a spectrum of intellectual decline with mild to severe cognitive functional deterioration [23]. Some of the researchers used the terms mild cognitive impairment (MCI) and dementia to represent cognitive impairment [24–27]. MCI is a transitional phase between healthy aging and dementia. It involves impairment in memory domains that was reported by stroke survivors or their caregivers. Other cognitive domains normally remain intact and stroke survivors have the ability to perform activities of daily living (ADL). In contrast with MCI, dementia occurs where patients experience severe memory loss, impairments in other cognitive domains, and decline in physical function [24, 25]. Statistics showed that approximately 1–25% of older adults with MCI per year are at high risk to develop Alzheimer's disease [28, 29]. Meanwhile, progression rate of MCI to dementia in 4-year period is 12% per year in stroke survivors as compared to progression rate of normal adults to dementia with only 1-2% per year within 10-year period [29]. This is a concern and calls for prevention of further deterioration as stroke will cause more severe cognitive damage.

In a study by Sachdev et al. [27], it was found that 58% of stroke survivors had cognitive impairment with a quarter of them diagnosed with dementia. It has also been reported that a stroke will double the risk of dementia [30]. In a Latin American study, 66% and 61% of stroke survivors were cognitively impaired at three and 12 months of the study (39% cognitive impairment with no dementia; 22% demented) [31]. The prevalence of cognitive impairment remains 21% at 3 months after stroke and after 14 years of follow-up period [32]. Prevalence varied due to differences in study population with nonlacunar and lacunar stroke being common in hospital and community based studies, respectively [33]. There is higher cognitive decline after lacunar stroke due to pathological causes where small vessel disease (SVD) affects wide region of the brain compared to nonlacunar stroke that involves extracranial region [33].

A cross-sectional study in United Kingdom (UK) found that the prevalence of cognitive impairment was high in the first month after ischemic stroke. This impairment involved speed and attention, frontal executive, nominal skills, perceptual skills, and visual memory [38]. Hurford et al. [38] reported that stroke survivors had improvement in visuospatial neglect and persistent impairment in speed and attention domain after 3 months of stroke. In a range of studies, researchers found that prevalence of cognitive impairment ranged from 7.5% to 72% from different studies which involved stroke survivors who had dementia and cognitive impairment with no dementia (CIND). The common terms for cognitive impairment that are used to refer to poststroke are cognitive impairment (PSCI), mild cognitive impairment (MCI), CIND, vascular cognitive impairment (VaCI), vascular mild cognitive impairment (VaMCI) subjective cognitive impairment (SCI), age-associated cognitive decline (AACD) dementia, vascular dementia (VaD), poststroke dementia (PSD), and cognitive dysfunction.

## 2. Methodology

**Figure 1:**
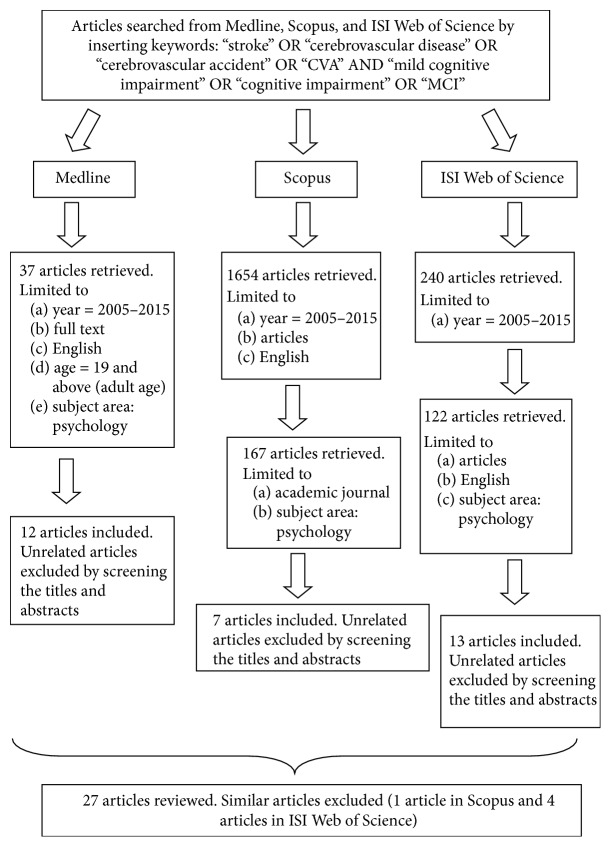
Flow chart of the reviewing process.

Searches were conducted using three databases (i.e., Medline, Scopus, and ISI Web of Science). Researchers used the same search terms for all databases: “stroke” OR “cerebrovascular disease” OR “cerebrovascular accident” OR “CVA” AND “mild cognitive impairment” OR “cognitive impairment” OR “MCI”. There were 37 articles in Medline, 1654 articles in Scopus, and 240 articles in ISI Web of Science. Searches were refined by identifying the studies published in the years 2005–2015, full-text English articles, and adult aged as studied population. In addition, researcher restricted searching to area of study in psychology as an additional search criterion. Three hundred and twenty-six retrieved articles were screened using their titles and abstracts. Only 32 articles were related to review objectives and reviewed (12 articles in Medline, 7 articles in Scopus, and 13 articles in ISI Web of Science). Articles related to prestroke cognitive impairment, studies on validation of measurement tools, and molecular factors among stroke survivors were excluded from this review. Only 27 articles were included in this review after excluding five similar articles from different databases (Figure 1).  Table 1 depicts the following features of related studies: (a) study reference, (b) objective of study, (c) sample size, (d) methodology, (e) setting, (f) prevalence of CI, and (d) outcomes (i.e., demographic, clinical, psychological, and physical).

## 3. Results

A total of 27 studies fulfilled this review's inclusion criteria in identifying demographic, clinical, psychological, and physical related risk factors that are likely to influence cognitive function among stroke survivors (refer to Table 1). Most of the studies (18 studies/67%) were cross-sectional in manner [17, 27, 34, 37–39, 41–50, 54, 55] and 74% (20 studies) were carried out in hospital settings [27, 35, 37–39, 41, 42, 44–46, 48–57]. Only three studies covered all the related risk factors in this review [35, 45, 46], whereas other studies discussed some parts of it. Eighteen articles included demographic factors in the discussion mainly age, education level, sex, marital status, life styles, family history, and socioeconomic status [17, 27, 32, 34, 35, 37, 40, 42, 44–46, 49, 51, 52, 54–57]. Seventeen studies conferred the influence of clinical factors including medical, vascular, and neurological factors on cognitive impairment [17, 27, 32, 35, 37, 40, 42, 44–46, 49, 51, 52, 54–57]. Depression was the main psychological factor of interest among researchers in 9 of the articles with psychiatric illnesses and stress as the most often mentioned, in spite of different psychological scales used in the studies [27, 35, 41, 44–46, 54, 55, 57]. Meanwhile, seven studies predicted the influence of physical functioning on cognitive impairment among stroke survivors [35, 36, 39–41, 45, 46].

### 3.1. Demographic Factors and Cognitive Impairment

One of the main predictors for cognitive impairment in stroke survivors reported in most studies was increased age [17, 27, 32, 34, 35, 37, 40, 45, 49, 51, 52, 54, 57]. Meanwhile, a lower level of education was highlighted as a risk factor for cognitive impairment in half of the studies [17, 34, 37, 42, 45, 46, 49, 50, 54]. Older stroke survivors with lower education level had higher prevalence of having cognitive impairment and history of stroke accelerated the development of dementia in them [34]. Life styles and dietary factors were also reported as risks for cognitive decline in stroke survivors. Four Asian studies conducted at hospital and community settings in China found that increased alcohol intake, lack of hobbies, longer sleep, irregular health check-up, and less vegetables, fruits, milk, and tea intake were identified as potential risk factors of poststroke cognitive impairment [17, 37, 54, 56].

Gender, family history of dementia, smoking habits, socioeconomic status, marital status, and ethnicity affected cognitive functioning [17, 27, 32, 34, 35, 42, 45, 46, 51, 52, 55]. In spite of the decline in cognitive status, approximately, 8% of stroke survivors improved from demented to nondemented state and 80% had stable cognitive functions after 24-month period [57]. Stroke survivors who had progressed to dementia were noted to be older, had history of cognitive impairment, and were prescribed with more medications [57]. Cognitive impairment was also high among black compared to white ethnic group. Meanwhile, manual workers were at a higher risk in comparison to nonmanual workers (UK occupational codes) [32].

### 3.2. Clinical Factors and Cognitive Impairment

Studies showed that stroke survivors who had medical conditions, such as low premorbid intellectual ability, preexisting cognitive impairment, and high stroke severity, and history of stroke and transient ischemic stroke (TIA), poststroke dysphasia, and urinary incontinence (aconuresis) had higher risk of cognitive decline [17, 27, 34, 35, 37, 44, 51, 52, 54, 57]. Vascular factors contributed more than medical factors as the predictors were heart disease, diabetes mellitus, atrial fibrillation, hyperlipidemia, TIA, obesity, high homocysteine level, presence of cerebral microbleeds, low diastolic pressure, hypotension, dementia, hypertension, and hyperlipoproteinemia that appeared as significant determinants in multivariate analysis [17, 30, 35, 45, 46, 51, 52, 54, 56, 57].

Neuroimaging characteristics which explained neurological factors of stroke were also the most influential determinants for poststroke cognitive impairment. There were nine studies reporting on neurological factors. Researchers indicated that infarct volume, left carotid infarction, high level of paraventricular white matter lesion (WML), brain atrophy, basal ganglia infarct, large infarct volume, more cerebral white matter hyperintensities (WMHs), cortical atrophy, lacunar infarct, small vessel occlusion, and dominant hemispheric lesions and cortex lesion were the significant risk factors for cognitive impairment [27, 32, 37, 44, 45, 49, 54]. On the other hand, two studies that provided neuroimaging information deduced that area of infarction (i.e., cortical, subcortical), type of infarctions (i.e., single, multiple), and size of lesion were found not to be correlated with frontal executive dysfunction and cognitive status [48, 50]. A study by Sachdev et al. [27] showed that stroke survivors who were in VaMCI group had larger volume of total, deep, and periventricular WMHs. It was also found that VaMCI group had smaller amygdala volume than noncognitive impaired (NCI) and control groups [27]. Besides that, a few researchers studied biomarkers as potentials to predict cognitive functional status. The authors showed that blood plasma homocysteine,* APOE ɛ*4 genotype, HbA1c, LDL, and HDL were clinical markers for cognitive status [44, 45, 47].

### 3.3. Psychological Distress and Cognitive Impairment

The common psychological distress among stroke survivors regardless of the severity of cognitive impairment was depression [35, 45, 47]. Depressive symptoms were reduced over time from time of admission to 6 months after stroke in nearly half of the stroke survivors with decrement in cognitive impairment [35]. In contrast, del Ser et al. [57] reported that depression does not associate with cognitive decline at 3-month period after stroke. It was found that four studies showed depression and psychological problems had some significant effects as risk factors for cognitive impairment [35, 46, 54, 55]. Inconsistent findings were reported in other studies [27, 44, 45, 57].

### 3.4. Physical Function and Cognitive Impairment

Stroke survivors who are physically dependent and more impaired usually perform poorly in tasks which require higher-order cognitive functions such as motor control, organization, problem solving, and memory. Lower performances in cognitive functions were positively associated with dependency in activity of daily living (ADL) [58]. It is a main concern after stroke because impairment in information processing is related to control of motor movement in executing ADL [59–61] Those who had intact prestroke cognition were reported to have higher personal ADL (P-ADL) and Barthel Index (BI) score compared to those who had cognitive impairment [36]. Stroke survivors with impaired cognitive impairment are highly dependent on instrumental ADL (I-ADL) which requires more complex tasks such as cooking, housework, and outdoor mobility in comparison to personal activities (i.e., eating, continence, bath, and dressing) [39].

Meanwhile, Liman et al. [40] reported differently where stroke severity was measured using Barthel Index score and was a significant predictor of cognitive impairment after 3 months of stroke. Stephens et al. [41] found that different impairment in cognitive domains would affect physical abilities differently. Researchers explained that those who had impairment in (1) reaction time would face disabilities in basic self-care, (2) impairment in executive function is associated with disabilities in intermediate self-care, (3) impairment in MMSE is associated with complex self-management, and (4) deficit in memory is associated with any basic, intermediate, and complex ADL components. Self-care activities were the main outcome measures examined because self-care activities correlate with general cognitive performance such as visual perception and visuomotor organization [43]. Other studies also found that stroke severity and lower Barthel Index score were significant predictors for poststroke dementia and other types of cognitive impairment [35, 45, 50].

## 4. Discussion

Findings from the current review suggested that there were various risk factors of cognitive impairment among stroke survivors. In terms of sociodemographic factors, age and education level were found to be the main predictors of cognitive impairment. Older adults were severely impaired in cognition due to the nature of stroke that accelerates cognitive decline [34]. It is deduced that stroke survivors with higher education level were less likely to be affected in their cognitive functioning because they have larger brain reserve capacity which can compensate for the brain damage [62].

Clinical factors were also main risk factors of cognitive impairment in stroke survivors. History of hypertension and diabetes may reduce the brain volume and also cause white matter lesions [63]. Affected regions of brain and types of stroke were also important variables which determined cognitive status of stroke survivors because lacunar infarcts and cortical strokes (i.e., parietal, cingulated, premotor, occipital, and temporal cortex) caused more severe deficits in cognitive status [64, 65]. More severe outcomes were noted when the stroke occurred at the important regions of brain which controlled vital functions.

In a study on the effects of blood pressure, low diastolic blood pressure and episode of hypotension were significantly associated with cognitive declines [57]. In contrast, a study conducted among MCI patient with high blood pressure showed that stroke survivors' performance deteriorated in tasks requiring rapid responses and set shifting (TMT A and B) and in expressive language (naming test) [66]. Therefore, it is questionable if hypertension leads to hemorrhagic stroke and vascular cognitive impairment. In addition, vascular cognitive impairment occurs due to impairment in blood-brain barrier and white matter lesion and increase in vascular permeability due to endothelial cell retraction which may also involve the blood flow [67]. Cerebral microbleeds in stroke survivors with lacunar infarcts had significant correlation with cognitive impairment. According to Zhang et al. [68] thalamic microbleeds reduced the orientation subscore, lobar (cortex/subcortex) microbleeds caused low visuospatial/executive subscores, and basal ganglia microbleeds were found to significantly reduce attention and visuospatial/executive subscores. The damage not only appeared at the affected area but also may disrupt other tissues nearby possibly due to lack of blood supply. Thus, pathological causes of infarction and tissue necrosis may have significant effect on cognitive function at the affected region [69].

Stroke had a significant impact on psychological wellbeing of stroke survivors. Stroke survivors usually experience sleep disturbances, low motivation, low self-esteem, and worries about their future due to restrictions and disabilities [70]. These psychological changes and stressful situations lead to depression and anxiety symptoms which affect their performance in executive function, memory, speed, and motor processing [71]. In addition, older adults who were highly anxious presented with cognitive decline and Alzheimer's disease after a year of cognitive impairment [72].

Mood changes and chronic stress due to physical limitations, restrictions in ADL, and low motivation were possible factors that caused emotional distress among stroke survivors [73]. Depression was believed to reduce the effort in cognitive test in older adults and resulted in poorer performance in the test [58]. This is due to declined cognitive processing which is related to slowness in responses and having difficulties to keep track in conversation. On the other hand, depression, somatisation, and insufficiency of thinking and acting were the most frequent psychiatric symptoms which were reported to have problem in executive and global cognitive function (i.e., orientation, attention, praxis, and language) [20]. Severe cases of cognitive impairment such as vascular dementia were also reported to present with more psychiatric symptoms than the milder ones [20].

Poor performance in executive function can affect individual's ADL and increase their risk for poststroke disability [74]. In addition, cognitive impairment also disrupts their orientation, perception, thinking process, and memory leading to dependence in self-care activities [43]. Stroke survivors with higher functional impairment were likely to be diagnosed with cognitive impairment with no dementia (CIND) [35].

There were two factors which caused cognitively impaired stroke survivors to have poor functional outcomes and these include higher stroke severity and poorer compliance to rehabilitation [75]. Assessment of cognitive status at initial stage of stroke is very important to determine functional outcomes. Physical performance of stroke survivors after 6 months of stroke was associated with cognitive impairment on admission and also cognitive improvement over 6 months [22]. This indicates that physical function was independently associated with cognitive status which was the main variable affected after stroke.

Identifying poststroke risk factors for cognitive impairment at early stage is important. Even more important is to evaluate the contribution of each risk factor towards cognitive impairment. Furthermore, current existing literature is lacking on integrating risk factors which increases poststroke risk for cognitive impairment. Previous studies mainly focus on identifying and grouping risk factors (e.g., [45, 76, 77]) rather than providing reliable information or data in predicting and reducing the risk for poststroke cognitive impairment. Future studies should focus on developing research on identifying and enhancing protecting factors against poststroke cognitive impairment. In addition, researchers need to explore combined effect of both pharmacotherapy and nonpharmacotherapy, especially psychosocial intervention to improve poststroke cognitive function. There is also a need for future research to focus on clinical and evaluative studies on holistic intervention to establish clinical effect size, especially on modifiable risk factors. The impact of modifiable risk factors on nonmodifiable risk factors to develop cognitive impairment needs more systematic research approach.

## 5. Conclusion

This review indicated that age, level of education, history of stroke, diabetes mellitus, hypertension, types of stroke, affected region, size and location of infarction, depressive symptoms, and physical function were the potential factors that determine cognitive status of stroke survivors. Identifying these risk factors would be beneficial for clinician and healthcare practitioners in the management of stroke survivors. This will assist in the prevention of further cognitive decline and improve psychological wellbeing through effective intervention. Further research examining in-depth and multiple risk factors discussed in this review is warranted.

## Figures and Tables

**Table 1:** Studies on cognitive impairment among stroke patients.

Study	Objective of study	Sample	Methodology	Setting	Prevalence	Outcome			
						Demographic	Clinical	Psychological	Physical
Sachdev et al. [27]	(a) Identify determinants for CI (b) Identify clinical features associated with development of VaMCI and VaD	169	Cross-sectional study	Hospital based	58% CI (VaD = 21.3%; VaMCI = 36.7%) Tools used to assess CI: (a) MMSE (b) Neuropsychological assessments (c) Consensus diagnosis by experts	(i) Older age (S) (60 years as cut-off) (ii) Lower education (NS) (iii) Female (NS)	Medical factors: (a) Lower NART-IQ (S)^*∗*^ (premorbid intellectual ability) (b) Higher IQCODE (S)^*∗*#^ (preexisting CI) (c) Higher ESS (S) (stroke severity) (d) Previous stroke (NS) (e) Previous TIA (NS) Vascular factors: (a) Homocysteine (NS) (b) Higher CVRF (NS) Neurological factors: (a) Infarct volume (S)^*∗*^ (b) Laterality of stroke (NS) (c) Cerebral hemisphere (NS) (d) Lacunar infarct (NS) (e) Brain atrophy (NS) (f) Number of infarcts (NS) (g) Higher WMH score (NS)	Depression (NS) Tools used: (a) GDS (b) HDRS	NR Tools used: (a) SOFAS (b) ADL (c) I-ADL

De Ronchi et al. [34]	Detect the impact of stroke on the occurrence of dementia and CIND in different age, sex, and education	7930	Cross-sectional study	Population based	11.6% CI (CIND = 5.1%; M: 4.1%; F: 5.7%) (dementia = 6.5%; M: 4.9%; F: 7.5%) Tools used to assess CI: (a) MMSE and Global Deterioration Scale (b) DSM-III-R and experts opinion	(a) Age and education modify effect of stroke on dementia (the risk was twofold stronger in older (75+ years old) and young people (61–75 years old) who had low education (0–3 years of schooling) with stroke as compared with higher education (4+ years of schooling) with stroke) (b) Sex did not modify the effect of stroke on dementia	History of stroke increased the risk ratio for dementia and CIND	NA	NA

Saxena et al. [35]	Determine the prevalence of depressive symptoms and cognitive impairment in stroke patients at 3 phases of rehabilitation processes	200	Observational study	Hospital based	Admission = 54.5% CI Discharge = 33.7% Follow-up (6 months) = 40.4% Tool used to assess CI: (a) AMT	(a) Older age (S)^*∗*^(≥76 years) (b) Divorced (S)	Medical factors: (a) Poststroke dysphasia (S)^*∗*^ (b) Poststroke urinary incontinence (S) (c) Cognitive impairment on admission (S)^*∗*#^ Vascular factor: (a) Ischemic heart disease (S)	Depressive symptoms on admission (S)^*∗*^ Tool used: (a) GDS	Severe physical functioning (S) Tool used: (a) BI

Cederfeldt et al. [36]	Examine the differences in performance of P-ADL in relation to cognitive impairment at pre- and postdischarge	45	Longitudinal study	Hospital based & community based	Acute phase = 29% CI 6 months = 20% CI 12 months = 10% CI Tool used to assess CI: (a) MMSE (b) CIMP-QUEST (c) Neuropsychological battery	NA	NA	NA	(a) Intact cognition (before & after): improved P-ADL (b) Impaired cognition (before & after): nonimproved P-ADL Tool used: (a) BI

Zhou et al. [37]	Identify the frequency and risk factors of cognitive impairment after stroke	434	Cross-sectional study	Hospital based	Poststroke CI = 37.1% Stroke-related CI = 32.2% First-ever stroke CI = 29.6% Tool used to assess CI: (a) MMSE (b) IQCODE	Personal factors: (a) Older age (S) (8.5 years older than cognitive intact group) (b) Lower education (S) (≤6 years) Life style factor: (a) Everyday alcohol drinking (S)^#^	Medical factors: (a) History of stroke (S)^#^ (b) Dysphasia (S) Neurological factor: (a) Left carotid infarction (S)	NA	NA

Hurford et al. [38]	Examine domain-specific patterns of cognitive change after ischemic stroke	209	Cross-sectional study	Hospital based	Changes in the prevalence of cognitive impairment in each cognitive domain from less than 1 month to over 3 months:				
					(a) Speed and attention: 72.4% to 37.9%				
					(b) Frontal executive function: 34.4% to 16.2%				
					(c) Nominal skills: 30.2% to 8.1%				
					(d) Perceptual skills: 29.5% to 8.1%				
					(e) Visual memory: approximately 18% to 10%				
					(f) Verbal memory: approximately 28% to 18%				
					Tool used to assess CI:				
					(a) Neuropsychological tests battery				
					No determinants on demographic, clinical, psychological, and functional data were reported				

Claesson et al. [39]	Explore the impact of cognitive impairment on ADL performances, utilisation, and costs of health care	149	Cross-sectional study	Hospital based	CI = 72% (dementia: 28%) Tool used to assess CI: (a) Rating of cognitive symptoms (b) DSM III-R	NA	NA	NA	Cognitively impaired: high dependency in I-ADL (i.e., continence, indoor mobility, toilet management, transfer, dressing/undressing, grooming, cooking, bath/shower, housework, and outdoor mobility) Tool used: (a) BI (b) SI

Liman et al. [40]	(a) Determine the frequency and predictors of CI (b) Examine the prevalence and factors associated with cognitive recovery and cognitive stability	630	Longitudinal study	Population based	(a) CI at 3 months = 14.8% (b) CI at 1 year = 13.3% (c) CI at 3 years = 11.8% Tool used to assess CI: (a) MMSE	(a) Predictor at 3 months after stroke: Older age (S) (b) Predictor for cognitive stability up to 3 years: Younger age (S)	(a) Predictor at 3 months after stroke: Diabetes mellitus (S)^#^	NA	(a) Predictor at 3 months after stroke: stroke severity on day 7/BI (S) (b) Predictor for cognitive stability up to 3 years: less stroke severity/high BI (S) Tool used: (a) BI

Stephens et al. [41]	Determine the relationship between attention, executive performance, and memory impairments with ADL impairments	339	Cross-sectional study	Hospital based	MCI = 19% CIND = 26% AACD = 44% Tool used to assess CI: (a) CAMCOG (b) CRT task performance (c) DSM-III-R (d) Experts' opinion	NA	NA	Depression is positively correlated with 18 BADLS items Tool used: (a) GDS	(a) CRT = disabilities in basic self-care^+^ (b) Executive function = disabilities in intermediate self-care^++^ (c) MMSE = disabilities in complex self-management^+++^ (d) Memory impairment: not associated with any disabilities in ADL Tool used: (a) BADLS

Tang et al. [42]	Determine the relationship between CMBs and CIND reversion	328	Longitudinal study	Hospital based	Impairment at baseline: (a) Visuomotor speed = 60.6% (b) Executive function = 49.6% (c) Visual memory = 29.9% (d) Verbal memory = 29.1% (e) Visual construction = 20.5% (f) Language = 13.4% (g) Attention = 6.3% Tools used to assess CI: (a) Modified VDB (b) FAB (c) MMSE (d) DSM-IV	Determinant of reversion of CIND: (a) Younger age (S) (b) Higher education (NS) Determinant of reversion of language domains: (a) Younger age (NS)	Determinants of reversion of CIND: (I) Medical factors: (a) Less visual constructional impairment (NS) (b) Less verbal memory impairment (NS) (c) More likely having visual memory impairment (NS) (d) More likely having executive function impairment (NS) (II) Vascular factors: (a) Absence of CMBs (S)^#^ (b) Absence of lobar CMBs (NS) (c) Hypertension (NS) (III) Neurological factors: (a) Small volume of WMHs (NS) Determinants of reversion of language domains: (a) Absence of CMBs (NS) (b) Small volume of WMHs (NS)	NA	NA

Akbari et al. [43]	(a) Investigate whether test performance in neurological and cognitive areas is able to predict daily task performance (b) Examine whether the potential of tool measures can predict functional outcomes	27	Cross-sectional study	Population based	NR Tool used to assess cognitive status: (a) LOTCA	(a) Stroke severity (i.e., motor impairment) correlates with dependency in ADL performance (b) Self-care activities correlate with general cognitive performance (total score of LOTCA) (c) Visual perception and visuomotor organization correlated with BI components (d) Determinants on demographic, clinical, psychological, and physical data were not reported (e) LOTCA is not suitable to predict dependency in BADLS performance after stroke Tool used: (a) Neurological impairment = NIHSS (b) ADL performance = BI			

Narasimhalu et al. [44]	Determine neuroimaging measures (i.e., infarcts, WMH, and neurodegeneration) associated with subjective cognitive impairment (SCI) in cognitively intact patients with lacunar stroke	145	Cross-sectional study	Hospital based	SCI = 30.9% NCI = 69.1% Tools used to assess CI: (a) MMSE (b) MoCA (c) FAB	(a) Age (NS) (b) Education (NS)	Medical factors: (a) MMSE (S)^*∗*^ Vascular factors: (a) Diastolic BP (NS) Neurological factors: (a) Basal ganglia infarct (S)^*∗*#^ (b) Temporal lobe infarct (NS)	Depression (NS) Tool used: (a) PHQ-9	NR Tool used: (a) Modified Rankin score

Khedr et al. [45]	(a) Determine the relative frequency of first-ever PSD (b) Determine the risk factors of PSD (c) Determine the relationship between total Hcy level and PSD	81	Cross-sectional study	Hospital based	PSD = 21% (PSD in ischemic stroke: 76.5%, PSD in hemorrhagic stroke: 23.5%) Tools used to assess CI: (a) Neuropsychological tests battery (b) MMSE (c) CASI (d) WMS-R (e) DSM-IV	Personal factors: (a) Older age (S) (b) Lower education (S) (c) Family history of dementia (S) Life style factor: (a) Smoking (S)	Neurological factors: (a) Large size of infarction (S) (b) Lacunar infarct (S) (c) Dominant hemispheric lesion (S) Vascular factors: (a) Hypertension (S) (b) High Hcy level (S) (c) Atrial fibrillation (NS) (d) Ischemic heart disease (NS) (e) Carotid stenosis (NS)	Depression (NS) Tool used: (a) HDRS	Motor and functional disability (S) Tools used: (a) SSS (b) BI

Makin et al. [33]	Determine the factors associated with the progression of cognitive impairment after stroke	193	Longitudinal study	Hospital based	3 months: (a) Cognitive intact = 72% (b) CIND = 9.3% (c) Dementia = 18.6% Tool used to assess CI: (a) SPMSQ (b) IQCODE (c) DSM-IV (d) Experts' opinion	(a) Older age (S) (b) Polydrug used (S)	Medical factor: (a) Previous CI (IQCODE) (S) Vascular factors: (a) Low diastolic pressure on admission (S) (b) Hypotension during admission (S)^#^	Depressive symptoms: not associated with cognitive evolution (progress or no progress) Tool used: (a) CDR (b) CES-D	NR Tool used: (a) BI

Čengić et al. [46]	Analyze and compare motor and cognitive impairment in stroke patients at acute, subacute, and chronic phases	50	Cross-sectional study	Hospital based	CI at acute, subacute, and chronic phases = 12% Tools used to assess CI: (a) Modified MMSE (b) SKT	Personal factors: (a) Older age (NS) (patient's age under 75 years) (b) Lower education (S) (partially or fully completed elementary school) (c) Heredity (S) Life style and habits: (a) Smoking (S) (b) Alcohol drinking (S) (c) Obesity (S)	Vascular factors: (a) Hypertension (S) (b) Hyperlipoproteinemia (S) (c) Diabetes (S) (d) Heart disease (S)	Stress (S)	(a) Physically inactive (S) (b) Acute and subacute phases: better motor recovery and better cognitive status Tool used: (a) ESS

Douiri et al. [32]	Evaluate the prevalence of cognitive impairment after first-ever stroke up to 15 years	4212	Longitudinal study	Population based	(a) CI at 3 months = 24% (b) CI at 5 years = 22% (c) CI at 10 years = 18% (d) CI at 14 years = 21% Tool used to assess CI: (a) MMSE (b) AMT	(a) Older age (S) (b) Ethnicity (black peoples) (S) (c) Socioeconomic status (manual worker) (S)	Neurological factors: (a) Lacunar infarct (S) (b) Small vessel occlusion (S)	NA	NR Tool used: (a) BI

Knopman et al. [47]	Determine the association of history of stroke with the diagnosis of MCI or cognitive impairment	2050	Cross-sectional study with case-control	Population based	MCI = 10.9% Tool used to assess CI: (a) Neuropsychological tests battery (b) DSM-IV	(I) Association of stroke with MCI:			
						(a) History of stroke was associated with a higher risk of MCI (adjusted for age, sex, and education)			
						(b) Association between history of stroke and MCI subtypes (aMCI and naMCI) did not change when diabetes, coronary heart disease, *APOE* genotype, and hypertension were added to the model			
						(c) History of stroke was associated with both aMCI and naMCI, while *APOE* ɛ4 genotype was associated with aMCI only			
						(II) Association of stroke with cognitive domains:			
						(a) History of stroke was significantly associated with lower cognitive function in other domains (language, executive, and visuospatial) except memory			
						(b) The magnitude of the association was strongest for the executive function domain in unadjusted analyses			
						(c) Association was elevated about 2-fold for language and visuospatial domains after being adjusted for age, sex, and education			
						(d) Association of stroke with language, executive, and visuospatial domains did not change when diabetes, coronary heart disease, *APOE* genotype, and hypertension were added to the model			
						(e) *APOE* ɛ4 genotype was only associated with poor performance in the memory domain			

Sundar and Adwani [48]	(a) Assess cognitive dysfunction at 3 months after ischemic stroke (b) Assess frontal executive function using MMSE (c) Evaluate the degree and type of cognitive dysfunction in ischemic stroke subgroups	164	Cross-sectional study	Hospital based	Cognitive dysfunction = 31.7% (17.07% had been impaired on frontal executive functions only) Tool used to assess CI: (a) Modified Folstein's MMSE (b) FAB	(a) Memory was significantly and commonly affected in multi-infarct strokes as compared to single infarcts (b) Frontal executive dysfunction was not significantly different in single versus multiple infarcts and cortical versus subcortical infarcts (c) Number of infarcts did not appear to influence cognitive dysfunction at 3 months of stroke (d) Determinants on demographic, clinical, psychological, and physical data were not reported			

Jokinen et al. [49]	Explore the severity and location of WMHs as predictor of neuropsychological test performance	323	Cross-sectional study	Hospital based	Dementia = 14.6% Tool used to assess CI: (a) Neuropsychological test battery (b) DSM-III-R	Predictors of neuropsychological deficits: (a) Age (S) (b) Education (S)	Predictors of neuropsychological deficits: (a) Total infarct volume (S) (b) Cerebral WMHs (S)^#^ (c) Cortical atrophy (S)^#^ Predictors of WMHs: (a) Poor executive functions (S)^#^ (b) Poor speed of mental processing (S)^#^ (c) Poor visual memory (S) (d) Poor delayed recall of objects (S) (e) Poor visuospatial task (S) (f) Poor short-term memory (NS) (g) Poor story recall (NS) (h) Poor verbal conceptualization (NS)	NA	NA

Cao et al. [50]	(a) Identify the neuropsychological impairments (b) Identify the clinical characteristics related to cognitively impaired patients	40	Cross-sectional with case-control study	Hospital based	(a) Dementia = 12.5% (b) CI = 7.5% (c) Partial CI = 20% Tool used to assess CI: (a) MMSE (b) Neuropsychological tests battery	(a) Lower education level is positively correlated with cognitive performance (global/partial impairment)			
						(b) Token test, RPM, and AVLT delay and similarities were more often significantly failed tests by patients than control. However, these tests did not correlate with the number and site of lesions, ultrasound pattern, and neurological conditions			
						(c) No correlation between size, number and side of lesions within demented patients, globally or partially impaired patients, and etiological diagnosis of stroke			
						(d) Dementia and CI were associated with a lower BI score			
						Tool used:			
						(a) Daily activity abilities: BI			
						(b) Depression scale: SDS			

Mizrahi et al. [51]	Evaluate the relationship between diabetes and overall cognitive status in patients with ischemic stroke	707	Retrospective study	Hospital based	CI: NR Tool used to assess CI: (a) MMSE	(a) Older age (S) (b) Gender (female) (S)^#^	Medical factor: (a) Previous stroke (S) Vascular factors: (a) Dementia (S) (b) Diabetes mellitus (S)	NA	NA

Mizrahi et al. [52]	Evaluate the relationship between atrial fibrillation (AF) and overall cognitive status in patients with ischemic stroke	707	Retrospective study	Hospital based	CI: NR Tool used to assess CI: (a) MMSE	(a) Older age (S) (b) Gender (female) (S)^#^	Medical factor: (a) Previous stroke (S) Vascular factors: (a) Diabetes mellitus (S) (b) Dementia (S) (d) Atrial fibrillation (S)^#^	NA	NA

Tang et al. [42]	Examine the frequency and clinical determinants of poststroke cognitive impairment in Chinese stroke patients without dementia	179	Cross-sectional study	Hospital based	CI: 21.8% after 3-month stroke Tool used to assess CI: (a) MMSE	(a) Lower education (S) (b) Gender (female) (S)^#^	Medical factors: (a) NIHSS dysarthria score (S) (b) Urinary incontinence (S) Vascular factor: (a) Atrial fibrillation (S)^#^	NA	NA

Sachdev et al. [53]	(a) Investigate neuropsychological features of the VaMCI and its progression over 3 years among stroke patients without dementia (b) Investigate risk factors for VaMCI conversion to dementia (c) Examine relationship of MRI measures with conversion	104 patients; 84 controls	Longitudinal study	Hospital based	Dementia over 3 years: (a) VaMCI subject: 24.4% (b) NCI subject: 8.5% Tool used to assess CI: (a) Consensus diagnosis by experts based on cognitive domains, functional status, and vascular etiologies	(i) Clear determinants of progression did not emerge			
						(ii) Neuropsychological impairment at baseline tended to predict greater decline			
						(iii) Global cognitive and functional impairment at baseline may be of importance in predicting dementia			
						(iv) Converters and nonconverters of VaMCI to VaD did not differ by age, sex, education, burden of vascular risk factors, or structural changes in brain			
						(v) VaMCI group had more vascular risk factors and more white matter hyperintensities at baseline than the NCI and control groups			
						(vi) Neuropsychological factor: greater decline of logical memory in VaMCI group			
						(vii) MRI measures: stroke patients had larger volumes of total, deep, periventricular WMHs and smaller amygdala volume (VaMCI group)			
						(a) Tools used in neuropsychological assessments: WMS-R, WAIS-R, Boston Naming Test, TMT, SDMT, Western Aphasia Battery, and NART			
						(b) Tools used in medical and psychiatric assessments: SOFAS, ADL, I-ADL, ESS, GHQ, GDS, HDRS, and Neuropsychiatric Inventory			

Tu et al. [17]	(a) Explore the prevalence and effects of vascular cognitive impairment (VCI) among ischemic stroke patients (b) Provide a basis for prevention and treatment strategies	689	Cross-sectional study with control group	Community based	VCI: 41.8% (a) VaCIND: 32.1% (b) VaD: 9.7% Tool used to assess CI: (a) MMSE and MoCA (b) Criteria in NINDS and AIREN (c) Expert consensus	Personal factors: (a) Older age (S) (b) Low level of education (S) (c) Professional worker (S) (d) Living alone (S)^#^ Behaviour and life style: (a) High alcohol intake (S) (b) Lack of hobbies (S)^#^ (c) Longer sleep (S) (d) Irregular health check-up (S) Dietary habits: (a) Not eating fruit/vegetables (S) (b) Not drinking milk (S) (c) Not drinking tea (S)	Medical factors: (a) Family history of stroke (S) (b) Aconuresis (S) Vascular factors: (a) Hyperlipidemia (S)^#^ (b) TIA (S) Neurological factors: (a) High level of paraventricular WML (S) (b) Macroangiopathy disease (S) (c) Brain atrophy (S)^#^	NA	NA

Zhang et al. [54]	Examine the incidence, neuropsychological characteristics, and risk factors of cognitive impairment 3 months after stroke in China	577	Cross-sectional study	Hospital based	PSCI: 30.7% (a) Visual impairment = 22.4% (b) Executive impairment = 11.6% (c) Memory impairment = 10.4% (d) Attention impairment = 3.1% Tools used to assess CI: (a) MMSE and MoCA (b) FOM, RVR, BD, and DS in WAIS-R (c) Consensus criteria and experts' opinion	Personal factors: (a) Older age (S) (≥65 years old) (b) Low education level (S)^#^(<7 years)	Medical factor: (a) Stroke severity: 3-month poststroke (S) Vascular factor: (a) Obesity (S)^#^ (waist circumference = men ≥ 102 cm; women ≥ 98 cm) Neurological factor: (a) Cortex lesion (S)	Depressive symptom (S) Tool used to assess CI: (a) CES-D	NA

Narasimhalu et al. [44]	(a) Compare cognitive performance and quality of life (QOL) in stroke survivors and controls (b) Examine predictors for cognitive function and QOL	109	Cross-sectional with case-control study	Hospital based	CI = 17.4% Tool used to assess CI: (a) Modified MMSE	(a) Older age (NS) (b) Sex (NS) (c) Lower education (NS)	Medical factors: (a) Previous psychiatric illness (NS) (b) Stroke duration (NS) (c) Paresis (NS)	Psychiatric morbidity (S) Tool used to assess CI: (a) GHQ-30	NA

^*∗*^Significant determinant in multivariate analysis; ^#^strong predictor; ^+^basic self-care (i.e., transferring, dressing, hygiene, teeth cleaning, going to toilet, bathing, eating, mobility, and drink preparation); ^++^intermediate self-care (i.e., shopping, house/gardening, transport, and games/hobbies); ^+++^complex self-management (i.e., finances, oriented to time, use telephone, and communication).

S = significant determinant; NS = nonsignificant determinant; NR = not reported; M = male; F = female; NA = not available; P-ADL = personal activities of daily living; I-ADL = instrumental activities of daily living; WMHs = white matter hyperintensities; WML = white matter lesion; ADL = activities of daily living; BI = Barthel Index; CI = cognitive impairment; MCI = mild cognitive impairment; VaMCI = vascular mild cognitive impairment; SCI = subjective cognitive impairment; NCI = no cognitive impairment; PSCI = poststroke cognitive impairment; VaD = vascular dementia; ESS = European stroke scale; NART = national adult reading test; NART-IQ = national adult reading test-intelligence quotient; IQCODE = informant questionnaire for cognitive decline in the elderly; CVRF = cardiovascular risk factor; TIA = transient ischemic attack; CIND = cognitive impairment no dementia; VaCIND = vascular cognitive impairment no dementia; AACD = age-associated cognitive decline; BADLS = Bristol activities of daily living scale; CRT = choice reaction time; CMBs = cerebral microbleeds; BP = blood pressure; FAB = frontal assessment battery; SSS = Scandinavian stroke scale; MMSE = mini mental state examination; MoCA = Montreal cognitive assessment; PSD = poststroke dementia; Hcy = plasma homocysteine; SPMSQ = short portable mental status questionnaire; aMCI = amnestic mild cognitive impairment; naMCI = nonamnestic mild cognitive impairment; RPM = Raven's progressive matrices; AVLT = auditory-verbal learning test; BSRT = Babcock story recall test; DST-B = digit span test backward; GDS = geriatric depression scale; HDRS = Hamilton depression rating scale; AMT = abbreviated mental test; CIMP-QUEST = cognitive impairment questionnaire; CDR = clinical dementia rating scale; SI = Sunnaas index of ADL; CAMCOG = Cambridge assessment of mental disorder in the elderly; VDB = vascular dementia battery; FAB = frontal assessment battery; LOTCA = Loewenstein occupational therapy cognitive assessment; PHQ-9 = patient health questionnaire; CASI = cognitive abilities screening instruments; WMS-R = Wechsler memory scale-revised; CES-D = Center for Epidemiologic Studies depression scale; SKT = short cognitive performance test for assessing memory and attention; SDS = self-rating depression scale; NIHSS = National Institutes of Health Stroke Scale; TMT = trail making test; SDMT = symbol digit modalities test; SOFAS = social and occupational functioning scale; GHQ = general health questionnaire; NINDS = National Institute of Neurological Disorders and Stroke; AIREN = Association Internationale pour la Recherche et L'Enseignement en Neurosciences; FOM = Fuld object memory test; RVR = rapid verbal retrieval; WAIS-R = Wechsler adult intelligence scale-revised; BD = block design; DS = digit span; DSM = diagnostic and statistical manual; MRI = magnetic resonance imaging.
